# Protozoan phagotrophy from predators to parasites: An overview of the enigmatic cytostome-cytopharynx complex of *Trypanosoma cruzi*

**DOI:** 10.1111/jeu.12896

**Published:** 2022-03-08

**Authors:** Ronald Drew Etheridge

**Affiliations:** Department of Cellular Biology, Center for Tropical and Emerging Global Diseases (CTEGD), University of Georgia, Athens, Georgia, USA

**Keywords:** cytopharynx, cytostome, endocytosis, kinetoplastids, microtubule, myosin, phagocytosis, root fiber, SPC, *Trypanosoma cruzi*, trypanosomatid

## Abstract

Eating is fundamental and from this basic principle, living organisms have evolved innumerable strategies to capture energy and nutrients from their environment. As part of the world’s aquatic ecosystems, the expansive family of heterotrophic protozoans uses self-generated currents to funnel prokaryotic prey into an ancient, yet highly enigmatic, oral apparatus known as the cytostome-cytopharynx complex prior to digestion. Despite its near ubiquitous presence in protozoans, little is known mechanistically about how this feeding organelle functions. Intriguingly, one class of these flagellated phagotrophic predators known as the kinetoplastids gave rise to a lineage of obligate parasitic protozoa, the trypanosomatids, that can infect a wide variety of organisms ranging from plants to humans. One parasitic species of humans, *Trypanosoma cruzi*, has retained this ancestral organelle much like its free-living relatives and continues to use it as its primary mode of endocytosis. In this review, we will highlight foundational observations made regarding the cytostome-cytopharynx complex and examine some of the most pressing questions regarding the mechanistic basis for its function. We propose that *T. cruzi* has the potential to serve as an excellent model system to dissect the enigmatic process of protozoal phagotrophy and thus enhance our overall understanding of fundamental eukaryotic biology.

Obtaining energy from your environment is a clear biological imperative, and for protozoan parasites, that environment, unfortunately for us, is our own bodies. The development of targeted approaches to impede these microscopic predators’ ability to raid our cellular resources has long been the dream of those searching to find ways to combat innumerable parasitic diseases. Currently, however, we suffer from an acute lack of understanding of even the basic mechanistic underpinnings of endocytosis in countless species of protozoans, whether they are free-living or parasitic. One such medically important family of protozoan parasites transmitted by blood-sucking invertebrates, the trypanosomatids (class kinetoplastida), infect millions of people globally causing significant levels of morbidity and mortality in endemic areas. Human infections are dominated by three main species of trypanosomatids (*Trypanosoma cruzi*, *Trypanosoma brucei*, and *Leishmania* spp.) with *T. cruzi*, the etiological agent of Chagas disease (i.e. American trypanosomiasis), being the focus of this review.

*Trypanosoma cruzi* chronically infects an estimated 7 million people in the Americas, with an at-risk population of 70 million, making it the most severe parasitic disease of the Americas (Perez-Molina & Molina, 2018; World Health Organization, 2015). Despite its impact, Chagas disease remains underreported, understudied, and underfunded and continues to result in a yearly loss of more than 50,000 lives and 0.586 × 10^6^ disability-adjusted life years (Mathers et al., 2006). Even in the United States, the burden is significant, with estimates of those currently infected ranging from 238,000 to 347,000 (Manne-Goehler et al., 2016). Infection is lifelong and current therapeutics are not curative, often resulting in debilitating cardiomyopathy in one-third of those infected (Groom et al., 2017). Like other trypanosomatids that cause disease in humans, *T. cruzi* is characterized by having a dixenous (two-host) life cycle. Its definitive host (or vector), the triatomine insect (family Reduviidae), is considered to be the primary transmission vector for humans, although other known forms of parasite transmission are possible including oral infection, blood transfusion, organ transplantation, and congenital infection (Nickerson et al., 1989; Pelosse & Kribs-Zaleta, 2012; Zapparoli et al., 2022). The life cycle of this parasite can be divided into four major developmental stages each serving a particular role (summarized in Figure 1); in the insect vector, you find the actively replicating epimastigotes (green) and infectious metacyclic trypomastigotes (blue), while in the mammalian host, you find replicating amastigotes (red) and infectious trypomastigotes (blue; De Souza, 2002; Kariuki et al., 2019; Sousa, 1999). It should be noted, however, that intermediate stages distinct from these major developmental forms have also been observed (De Souza & Barrias, 2020). Natural transmission by the insect vector occurs during the course of a blood meal when the parasite, released in the vector’s excrement as a metacyclic trypomastigote (blue), enters the host bloodstream either through the bite wound or nearby mucous membrane. The excreted parasite is capable of invading any nucleated cell it encounters. Upon reaching the host cell cytosol, *T. cruzi* converts into its amastigote (red) form and replicates for ~3– 5 days until the host cell is completely filled with potentially over a hundred parasites. At this point, *T. cruzi* will elongate its flagellum and transform into infectious trypomastigotes (blue), lyse the host cell, and either initiate another round of host cell invasion or be taken up by a new insect vector where it will propagate as epimastigotes (green) and complete the transmission cycle (Barrett et al., 2003; Souza et al., 2010).

In order to complete their life cycle effectively, however, it is essential that parasites ensure unimpeded access to their host’s nutrient stores. To achieve this, the trypanosomatids have been shown to employ both integral membrane transporters, to bring in simple nutrients, and bulk endocytosis, to bring in larger more complex extracellular material such as proteins and lipid complexes (Marchese et al., 2018; Michels et al., 2021; Souza et al., 2009). It is worth noting that for these protozoans, this process of endocytosis can only occur at specialized regions of the plasma membrane (PM) that are not obstructed by the rigid microtubule cytoskeleton that covers most of the organism (Figure 3 gray lines; Morgan et al., 2002, 2002; Souza et al., 2008). To date, most of what we know of the endocytic process in this parasitic family has come from studies of a single species, *Trypanosoma brucei*, the causative agent of African trypanosomiasis (Overath & Engstler, 2004). This focus is due, primarily, to the relative ease of *in vitro* culture and the wide array of high-quality genetic tools, including robust RNAi-mediated gene knockdown, that are unavailable in the study of related trypanosomatids (Meissner et al., 2007). Unfortunately, *T. brucei* and members of the salivarian trypanosomatids (i.e. those transmitted through the mouth of biting insects) are far from representative of the global family of kinetoplastids, or even parasitic trypanosomatids for that matter, as they endocytose extracellular material *exclusively* at the membranous invagination known as the flagellar pocket (FP; Field & Carrington, 2009). All remaining free-living and parasitic kinetoplastids, it turns out, employ a completely different and far more ancient mechanism of nutrient uptake using an oral apparatus known as the cyto*s*tome-cyto*p*harynx *c*omplex or SPC for short. This feeding structure is analogous to a mouth (cytostome) and gullet (cytopharynx) and begins as a stable opening adjacent to the flagellar pocket that extends internally as a tubular invagination that carries endocytosed material to the posterior end of the cell for digestion (summarized in Figure 3; Adl et al., 2012; De Souza, 2002; Losinno et al., 2020). Due to historical limitations in the molecular tools available to study *T. cruzi*, investigators seeking to understand the SPC in trypanosomatids have been forced to rely heavily on the use of observational techniques such as electron microscopy (EM)-based freeze fracture, fracture flip, transmission (TEM), scanning (SEM), tomography, and focused ion beam SEM (FIB-SEM; Chiurillo & Lander, 2021; Lander et al., 2019). These techniques and the resulting high-quality structural data have revealed, with ever-increasing levels of detail, the remarkable complexity of this fascinating organelle (Alcantara et al., 2014, 2017, 2021; Chiari et al., 1978; De Souza et al., 1978; Girard-Dias et al., 2012; Martinez-Palomo et al., 1976; Milder & Deane, 1969; Okuda et al., 1999; Pimenta et al., 1989; Pitelka, 1961; Porto-Carreiro et al., 2000; Preston, 1969; Ramos et al., 2011; Souza, 1999; Souza et al., 1978, 2009; Steinert & Novikoff, 1960; Vatarunakamura et al., 2005; Vidal et al., 2016). Where this work has been found wanting, however, is in its inability to provide a clear mechanistic explanation for how the SPC *actually* brings in food. Our laboratory’s recently published work on SPC-targeted myosin motors has only just begun to elucidate the molecular machinery central to SPC function with an array of important questions still remaining to be answered (Chasen et al., 2020). Far from being comprehensive, our goals in this review are to briefly summarize our current conception of the *T. cruzi* SPC with regard to its evolutionary history and physical structure and use a variety of prior seminal observations in order to focus our attention on two broad questions which we think, when answered, will significantly enhance our understanding of how the SPC organelle functions as a whole: (1) *How is food captured at the surface? and* (2) *How does the endocytic machinery pull food in?* It is our hope that answers to these questions will not only bring us closer to a more complete view of the SPC in this important human pathogen, but also contribute more broadly to our understanding of basic protozoan biology and the uniquely eukaryotic process of endocytosis as well.

## WHERE DID THE SPC COME FROM? KINETOPLASTIDS, FROM PREDATORS TO PARASITES

**FIGURE 2:**
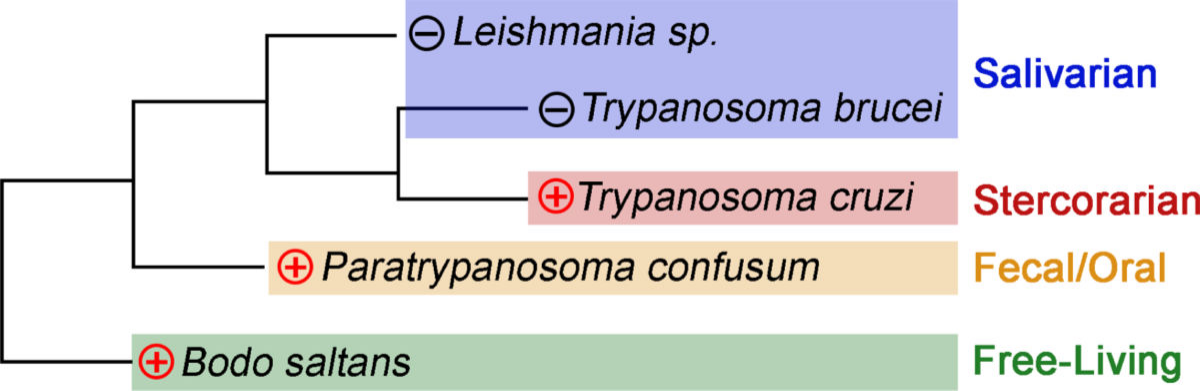
Phylogenetic analysis of SPC retention in kinetoplastids. Evolutionary relationship between the free-living and parasitic kinetoplastids. (+) branches include free-living *B. saltans* (green), monoxenous *P. confusum* (orange), and stercorarian *T. cruzi* (red) trypanosomatids maintained the SPC while (−) salivarians (purple) lost it. Tree adapted from Skalicky et al. (2017)

**FIGURE 3:**
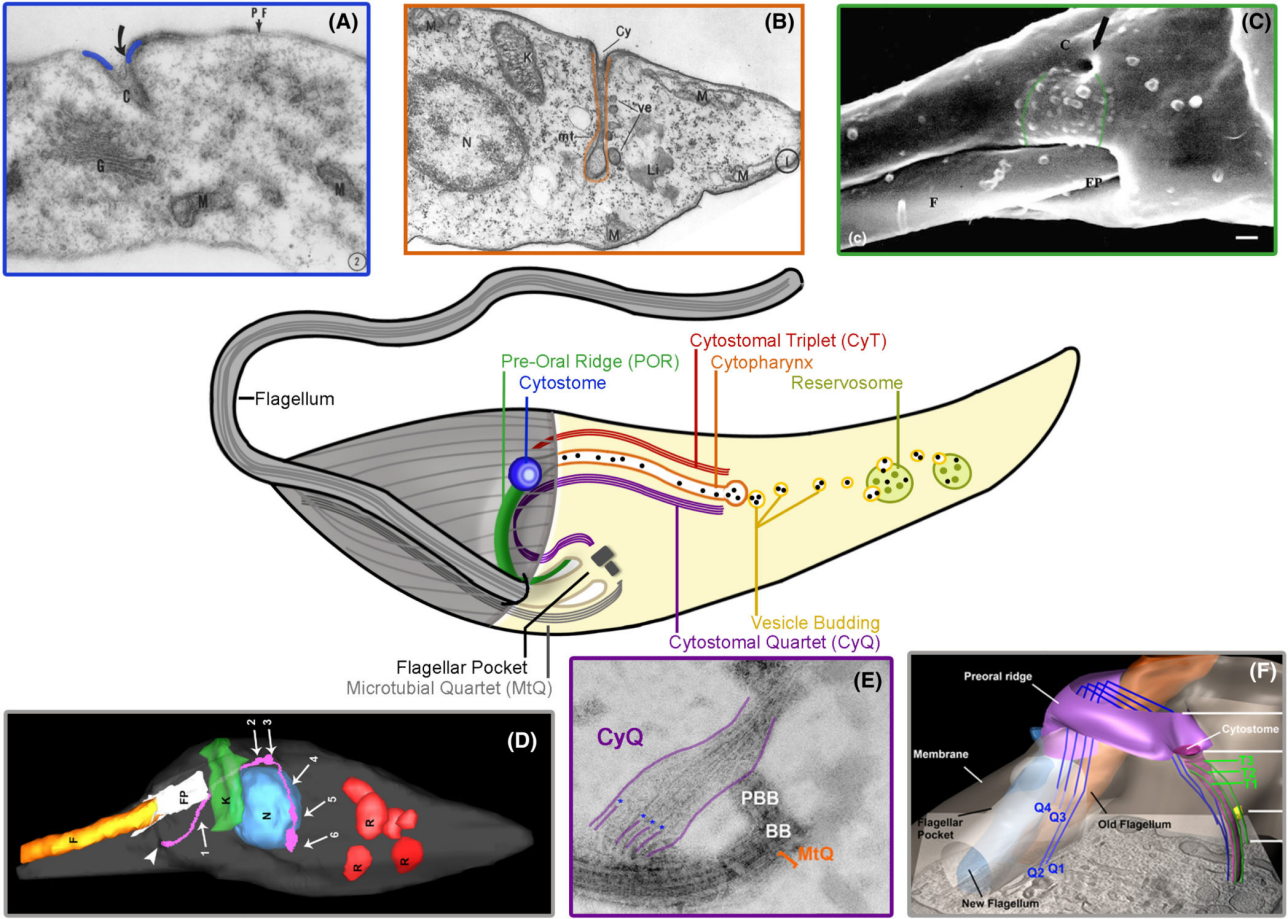
Structure of the cytostome-cytopharynx complex (SPC). Center: A schematic reference for the endocytic apparatus of *T. cruzi*: Preoral ridge (green), cytostome pore (blue), cytopharynx (orange), CyQ (purple), CyT (red). (A) First transmission EM (TEM) of a trypanosomatid (*T. mega*) SPC entrance (blue). Adapted from Steinert and Novikoff (1960). (B) TEM of the *T. cruzi* SPC cytopharynx (orange). Adapted from Milder and Deane (1969). (C) SEM of *T. cruzi* preoral ridge (green) and SPC entrance (arrow). Adapted from Nakamura et al. (2005). (D) 3D reconstruction of the *T. cruzi* cytopharynx (pink tubule). (E) Whole-mount EM of *T. cruzi* CyQ microtubules (purple lines). (F) 3D reconstruction of CyQ (blue) and CyT (green) of the *T. cruzi* SPC. Figures (D), (E), and (F) adapted from Alcantara et al. (2014)

Protozoal phagotrophy is an extremely ancient mode of nutrient acquisition and, with respect to the evolutionary history of eukaryotes, is considered to have been a necessary prerequisite for the acquisition of the endosymbiotic mitochondrial and photosynthetic organelles we see today (Cavalier-Smith, 2002). As a result, phagotrophy can be found in all of the major phylogenetic branches of the protistan family. It is estimated that phagotrophic predators, such as flagellates and ciliates, consume up to 75% of the daily phytoplankton production from oceanic ecosystems and, as such, play pivotal roles in carbon cycling within global microbial food webs (Sherr & Sherr, 2002). In addition to free-living predators, many of these protozoan phagotrophs also evolved into obligate human parasites and the kinetoplastids, an environmentally widespread and ecologically important group of single-celled flagellates are a clear example (Flegontov et al., 2013; Lukes et al., 2018; Skalicky et al., 2017). Due to the severe impact that kinetoplastid parasites continue to have on human health, it should be no surprise that the majority of what we know of this protozoan class has been gleaned from the study of its disease-causing members (Burza et al., 2018; Buscher et al., 2017; Perez-Molina & Molina, 2018). In order to better understand the basic biology of trypanosomatids, it is worth discussing their likely evolution from free-living aquatic excavates who used their cytostomes to hunt, capture, and ultimately digest bacterial prey (analogous process for *T. cruzi* shown in Figure 3; Lukes et al., 2014; Simpson et al., 2002). In an attempt to trace the evolutionary origins of this parasitic family, several studies have demonstrated that the bodonids, e.g. the biflagellate *Bodo saltans*, are modern-day representatives of free-living kinetoplastids. The diverse family of bodonids can be readily found in almost all marine and freshwater habitats and uses self-generated currents with one of their two flagella to filter feed on bacteria (Flegontova et al., 2018; Stevens, 2014). How this transition from bacterial predation to the parasitism we see in trypanosomatids today first occurred has been the source of contentious debate for almost a century, but with the discovery of *Paratrypanosoma confusum* as the earliest known basal branching monoxenous (one-host) trypanosomatid, it has become apparent that the initial transition to a parasitic lifestyle first began in arthropods and that dixenous parasitism likely arose independently on several occasions (Figure 2 adapted from Skalicky et al. 2017; Flegontov et al., 2013; Lukes et al., 2018; Simpson et al., 2002; Skalicky et al., 2017; Stevens, 2008). The colonization of the insect intestinal tract, it seems, proved advantageous as numerous examples of previously free-living organisms (including apicomplexans) are known to have taken up residence in the relatively stable ecological niche of the metazoan intestine (Harp, 2003; Sinha et al., 2012). It is in the arthropod intestine where these parasites presumably first perfected the fecal/oral route of transmission which, even today, is the dominant mode of monoxenous trypanosomatid dissemination. What has become clear from the analysis of the *P. confusum* genome is that during this first transition to a parasitic lifestyle, these organisms lost their 2nd flagellum and streamlined a number of metabolic pathways (Deschamps et al., 2011; Harmer et al., 2018; Opperdoes et al., 2016). Intriguingly, the fecal-transmitted parasites, like *P. confusum* and *T. cruzi*, retain many more ancestral genes and structures relative to the salivarian trypanosomatid clades and this includes the ancestral form of nutrient acquisition via the SPC endocytic organelle (Skalicky et al., 2017). It is worth noting that the presence of an SPC has been observed in *all* monoxenous trypanosomatids as well as in all the dixenous fecal-transmitted stercorarians studied to date, with only the salivarians (*T. brucei* and *Leishmania* spp.) completely discarding the SPC as an endocytic structure (Landfear & Ignatushchenko, 2001; Preston, 1969). Although we will discuss the question as to why the stercorarians may have selectively retained the SPC at the end of this review, it appears to correlate with some aspect of life within the insect vector intestinal tract itself.

## WHAT DOES THE SPC LOOK LIKE? HISTORICAL OVERVIEW OF THE STRUCTURE AND DYNAMICS OF THE SPC IN *TRYPANOSOMA CRUZI*

**FIGURE 4:**
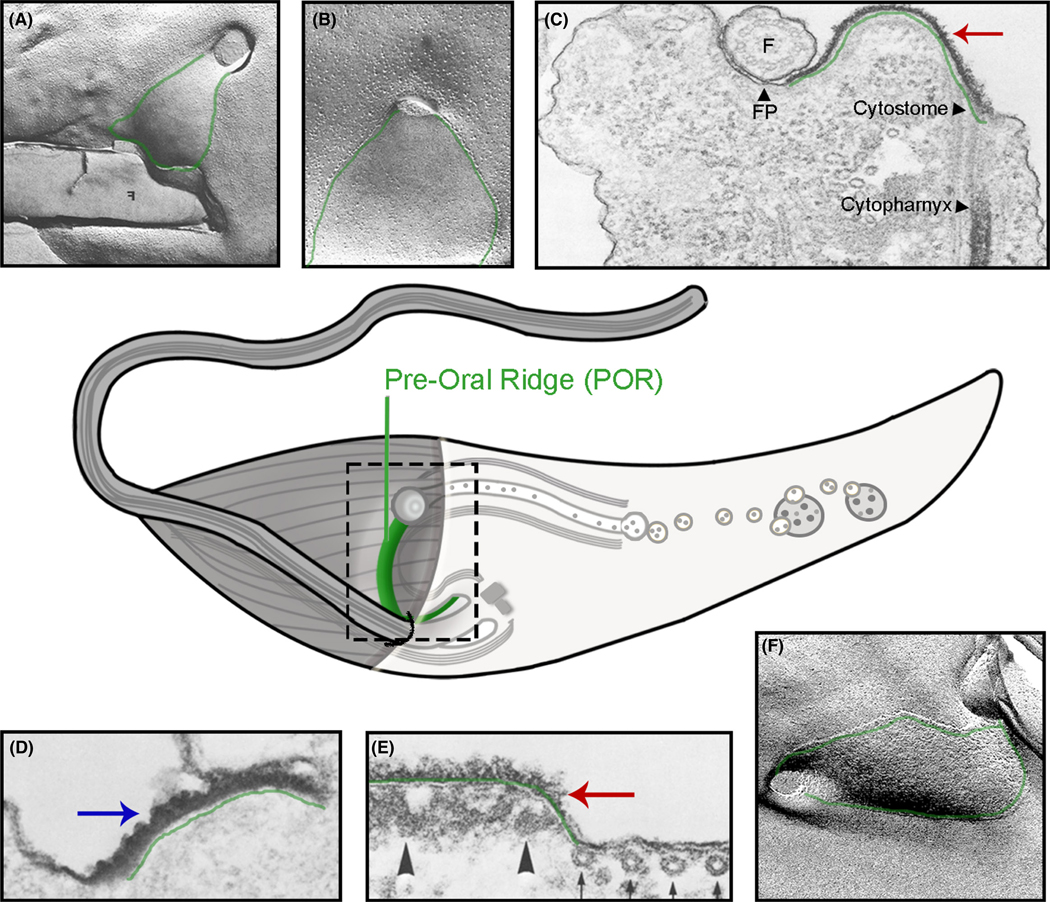
Composition of the *T. cruzi* preoral ridge (POR). Center: schematic focus on POR region in green. (A) Freeze-fracture EM of P-face of POR (green). (B) Higher magnification of freeze-fracture EM of P-face. POR outlined in green. (C) TEM and ruthenium red stain (red arrow) of *T. cruzi* POR (green). SPC (arrowheads). (D) TEM and ConA staining (blue arrow) of POR (green line). (E) TEM and ruthenium red stain (red arrow) of POR (green line). (F) Freeze-fracture flip EM of POR (green outline). Figures (A– E) adapted from Martinez-Palomo et al. 1976. Figure (F) adapted from Pimenta et al. (1989)

Our current understanding of the SPC structure in kinetoplastids is the result of a 60+ years journey beginning with work published in 1960 by Steinert and Novikoff who, by examining TEM images of the frog trypanosome *Trypanosoma mega*, gave us our first direct look at the cytostome in this protozoan family (Steinert & Novikoff, 1960). In this era, there were already a number of excellent observational studies of the oral apparatus of many ciliates and flagellates using both light and electron microscopic techniques and, due to the clear structural similarities, the authors applied the same cytostome nomenclature to this structure in kinetoplastids (Boeck, 1921; Corliss, 1959). This seminal work was able to show that the trypanosome cytostome was a stable opening (Figure 3A blue lines) present at the parasite surface that connected to a tubular invagination that was seemingly lined with “pellicular fibrils”, later shown to be microtubules. This study was also the first to note that tracer food, in this case, electron-dense ferritin, was curiously found only at the cytostomal mouth and nowhere else on the PM. This observation, it turns out, would be repeated numerous times over the subsequent decades and would lend support to the idea that surface receptors may also be present in these parasites, a topic we will discuss in greater detail in the next section. It was not, however, until the work of Milder and Deane in 1969 that we were able to get our first direct look at the SPC structure of *Trypanosoma cruzi* itself (Milder & Deane, 1969). This work put on full display the true complexity of this structure in what was the clearest view yet of the SPC’s internal organization. This was an important finding, in part, because by this time, many salivarian trypanosomes had already been examined via EM and because they lacked an SPC, it was generally assumed that this apparatus was going to be universally absent in mammalian trypanosomatids (Vickerman, 1969). As seen with *T. mega* years prior, a number of cytoplasmic vesicles were observed lying adjacent to the cytopharynx in a seemingly organized fashion (Figure 3B orange outline) with associated microtubules prophetically suggested to act as “…a sort of skeleton for the whole organelle…” In an attempt to explain how the SPC might function, the authors put forth the intriguing hypothesis that these microtubules might be contractile in nature and thus could potentially operate as a rudimentary pump. This idea of an overtly mechanical SPC was met with skepticism in subsequent work by T. M. Preston that same year who, through careful examinations of the fish trypanosome *Trypanosoma raiae*, generated the most detailed analysis of the SPC apparatus yet seen (Preston, 1969). In addition to again pointing out the preferential binding of protein ligands to the cytostome entrance, the proposed model of the SPC suggested that the previously observed cytostomal microtubules were not, in fact, part of the subpellicular array as originally suspected, but instead originated at the base of the flagellum (location of the basal body), wound up and around the flagellar pocket and then descended again into the parasite cytosol alongside the cytopharynx. Between five and six SPC-associated microtubules were seen and, due to their distinctive electron density, were noticeably different from the PM-associated subpellicular microtubules. At the conclusion of this work, the author also speculated on the central question of how the SPC might function by saying “The way in which fluid is circulated within the cytopharynx is not clear. In the absence of any evidence that the cytostome contracts and acts as a pump, it may be that the tip to base beating of the flagellum causes a current down one side of the flagellar canal, and that part of this current is deflected into the cytopharynx.” However, later work by Meyer and De Souza showing immotile amastigotes taking up host cytosolic melanin granules, appeared to make the necessity of flagellar currents to drive endocytosis less likely (Meyer & Souza, 1973). The field would have to wait until 1976 in work presented by Martinez-Palomo et al. for the next major advance in our understanding of the SPC (Martinez-Palomo et al., 1976). Although we will expound on the findings and implications of this work in greater detail in the next section, it is worth pointing out that this extensive freeze-fracture-based EM study of the *T. cruzi* PM revealed for the first time just how dramatically different the membrane surface adjacent to the cytostome entrance, later termed the preoral ridge (POR), was from the rest of the PM and highlighted the SPC’s intimate connection to the flagellar pocket membrane itself (Figure 4C green line). These freeze-fracture observations were, years later, nicely complemented in work by Nakamura et al. who, using newly improved techniques for SEM, provided the first direct look of the SPC from the parasite exterior (Figure 3C green outline), reinforcing the idea that the POR region was indeed a distinct membrane domain with a potentially important role in the endocytic process (Vatarunakamura et al., 2005). In addition to the structure of the SPC, follow-up studies by De Souza et al. released in 1978 sought to determine what the final destination was for endocytosed material. This analysis followed tracer protein, initially bound to the cytostome entrance, and observed movement down the cytopharynx and deposition into multivesicular structures, previously observed by Bretana and O’Daly in 1976, that later came to be known as the reservosomes (Bretana & O’Daly, 1976; Soares & De Souza, 1988; Souza et al., 1978). It is now generally accepted that the bulk of endocytosed material ends up in these prelysosomal reservosomes which serve as the main energy source for metacyclogenesis when parasites are faced with nutrient deprivation in the vector hindgut (Soares et al., 1989).

Research in the following decades provided a number of interesting insights into the nature of SPC function with debates persisting over what constituted receptor-mediated versus fluid-phase uptake and whether or not endocytosis occurs within the flagellar pocket of *T. cruzi* as well. The details of the SPC structure, however, would not be seriously revisited until 1999 when Okuda et al. examined the microtubule arrays previously shown to associate with the SPC (Okuda et al., 1999). By this point, extensive studies of the microtubule quartet (MTQ) of *T. brucei*, which is thought to template the location where the flagellum and cell body connect, had clearly shown them to originate at the basal body (Dong et al., 2020; Hoog et al., 2016; Vaughan & Gull, 2015). However, because *T. cruzi* also has an MTQ, it remained possible that the previously observed SPC microtubules which originate at the flagellar base were, in fact, simply the MTQ root fibers. In this report, EM was combined with flagellar isolation methodologies to produce whole mounts of the *T. cruzi* cytoskeleton revealing a stable, detergent-resistant association of the SPC and the flagellar/basal body structures. This connection was now conclusively shown to be based upon microtubules that were an independent array and distinct from the previously described MTQ.

At the time, it was difficult to imagine that additional insight into the SPC could be made with the available technology, but this lull would be finally broken in 2012 when Girard-Dias et al., using high-pressure freezing combined with serial electron tomography and 3D reconstructions, created an impressive high-resolution map of the internal structures of *T. cruzi*. This technique allowed researchers to clearly show that *four* SPC-specific microtubules originate at the flagellar pocket. Importantly, however, when this array was followed into the parasite cytosol, several additional microtubules seemed to spontaneously appear. In the end, the exact origin of these extra microtubules was not settled in this work, but, nevertheless, they were proposed to be the same five or six SPC microtubules observed by T.M. Preston over 40 years prior (Preston, 1969). Even though this work was unable to see the SPC in its entirety, it did show that it was now possible to produce a high-resolution reconstruction of the internal structure of these parasites. Within 2 years, Alcantara et al. used these same methodologies to provide the first definitive high-resolution 3D view of the entire endocytic apparatus of *T. cruzi* and revealed, with stunning clarity, the true nature of this cell spanning tubular invagination from start to finish (Figure 3D pink tubule; Alcantara et al., 2014). These results also settled several debates including questions regarding the microtubule root fibers that had emerged from the fragmentary data accumulated over the decades. We could now see that parasites in fact have *two* distinct sets of microtubule root fibers supporting the SPC structure. The first set, which had been observed by several groups, is specifically composed of four microtubules, referred to here as the cytostomal quartet or CyQ. The CyQ originates from the basal body complex, much like the MTQ, and winds up the flagellar pocket to the PM where a sharp curve in the quartet occurs prior to descending abruptly and traveling deep into the parasite cytosol. Unlike the fragmentary hints from prior TEM/whole-mount cytoskeletal analyses, this work clearly showed that it is the inherent curvature of the CyQ root fibers, present even after membrane extraction (Figure 3E purple outline), that produces the curved shape of the surface POR plasma membrane region. Significantly, the second rootlet set was now seen to be a completely independent array of three microtubules, referred to as the *cy*tostomal *t*riplet or CyT, which originate directly adjacent to the cytostome opening and track beside the CyQ to form a “gutter” shape around the cytopharynx (Figure 3F blue CyQ and green CyT lines). Using transferrin as a tracer, Alcantara et al. were also able to show that there is active membrane trafficking along the naked side of the cytopharynx tubule and not just at the terminating end. This work likely put to rest the perennial debate over endocytosis in the flagellar pocket, as no tracer-labeled vesicles were ever seen emanating from this region in any of the 3D reconstructions. From this foundational work, several follow-up reports from this group dissected how the SPC fully breaks down both when parasites differentiate into metacyclic trypomastigotes (Vidal et al., 2016) as well as when they transit through the cell cycle (Alcantara et al., 2017). It appears that the SPC undergoes a massive restructuring during the cell cycle as the cytopharynx tubule first begins to break down in early G2, followed by loss of the CyT and finally a shortening of the CyQ. As a result, in late G2/M, nearly all cells with a duplicated kinetoplast (i.e. 1N2K2F) no longer endocytose. After duplication of the flagellar pocket, however, the CyQ is reextended, followed by generation of the CyT and finally ending in invagination of the FP membrane to regenerate the entire SPC. Endocytic capacity is, therefore, only briefly stopped and quickly restored even before cytokinesis had been completed. Follow-up reconstructions of the endosomal system itself also provided a look at the endocytic network connecting the SPC to the final reservosome destination (Alcantara et al., 2018). Recently, Alcantara et al. completed the life cycle characterization of the SPC by focusing on intracellular amastigotes and were able to show that this structure was highly analogous to that seen in epimastigotes (Alcantara et al., 2021). In trying to understand the nature of endocytosis in *T. cruzi*, we are fortunate to be in possession of a wealth of structural data with which to begin developing testable models regarding how this organelle functions. Thanks to recent advances in molecular tools to modify *T. cruzi* genes, as well as increasingly well-annotated genomic databases, the field is well positioned to begin deciphering how this structure is built and regulated in order to hopefully answer the question as to why related parasites, such as *T. brucei*, were able to completely abandon the SPC as a mode of nutrient uptake (Lander et al., 2017; Peng et al., 2014; Wang et al., 2021).

## HOW IS FOOD CAPTURED AT THE SURFACE? THE MYSTERY OF THE PREORAL RIDGE AND THE ELUSIVE *T. CRUZI* RECEPTOR

Does *T. cruzi* use surface receptors to capture food and trigger endocytosis? This has been a long-standing question in trying to understand endocytosis in not only *T. cruzi*, but also in many species of protozoa that employ an SPC. In indirect studies of various free-living bacterivorous protozoans, it has been observed that the area adjacent to their cytostome is uniquely coated with what appear to be lectin-like surface receptors as well as mannose-d ecorated structures recognized by the lectin Concanavalin A (ConA; Roberts et al., 2006). Although speculative, it has been proposed that these carbohydrate-recognizing receptors may facilitate binding to the bacterial cell wall and thus enhance the overall efficiency of prey capture and uptake (Martel, 2009; Wootton et al., 2007). It is, therefore, within reason to consider that *T. cruzi* may continue to use versions of these ancestral surface receptors to seize nutrients directly from its host. Although a putative C-type lectin receptor was identified in the *T. cruzi* endocytic proteome, to date, there have been no published reports characterizing even a single-surface receptor for *T. cruzi* involved in either binding to or supporting endocytosis of cargo (Brosson et al., 2017). On the other hand, what we do have are reams of observational data showing that protein targets ranging from ferritin, horseradish peroxidase (HRP), bovine serum albumin (BSA), transferrin, and immunoglobulin (IgG) are readily taken up by the SPC (Alcantara et al., 2014; Chasen et al., 2019, 2020; Correa et al., 2008; Scott et al., 1997). What’s more, these proteins bind specifically to the specialized surface membrane of the preoral ridge (POR) with sufficient affinity to resist displacement following multiple washes. In fact, we have not identified a protein that *cannot* be bound by this region and it is this seemingly nonspecific binding or “sticky” quality of POR which makes the search for potential receptors so intriguing. To begin thinking seriously about how this specialized membrane area might bind cargo, it is worth considering the many unique qualities of the POR itself. As mentioned in the prior section, the first evidence which demonstrated just how different this POR membrane really was, came from several freeze-fracture and transmission EM experiments, carried out in 1976 by Martinez-Palomo et al. By freezing parasites and splitting them in such a way as to separate the plasma membrane bilayer, one could get a glimpse of the normally hidden internal face of both the extracellular (E face) and cytoplasmic (P face) leaflets. Using this technique, transmembrane (TM) proteins will appear as raised protuberances on the cytoplasmic P face and, due to being removed from the outer leaflet, as divots in the extracellular E face. Freeze fracture, therefore, revealed that the POR region connecting the cytostome to the flagellar pocket was so distinct that it had the appearance not unlike a “membrane river” flowing down into the SPC opening (Figure 4A green outline; Martinez-Palomo et al., 1976). Zooming in on this POR region (Figure 4B green outline), the authors highlighted two important phenomena; a clear paucity of TM domain-containing proteins *within* the POR membrane region, thus giving it a smooth appearance, and a linear “palisade” boundary of TM proteins lining the banks of this membrane river. This palisade is reminiscent of the cytoskeleton-driven “fence and picket” like structures that partition lipid raft domains in other eukaryotic systems to prohibit the free flow of TM proteins into this region (Ritchie et al., 2003; Saha et al., 2016). Using TEM and the polysaccharide stain Ruthenium red, the authors observed that the POR surface (underlined in green) was not only uniquely glycan rich when compared to the rest of the PM (Figure 4C and E red arrow), but it also extended *deep* into the normally unseen domain of the flagellar pocket itself (Figure 4C green line). Although not explicitly stated, this observation opened the possibility that the membrane being pulled into the SPC may originate from the flagellar pocket via vesicle fusion and thus provide a continuous source of endocytic membrane and surface receptor materials. The glycans observed by Ruthenium red staining also appear to be rich in mannose as the lectin ConA also preferentially labels the POR ridge not unlike what had been observed in free-living protozoa mentioned previously (Figure 4D blue arrow). This binding of ConA to the POR has since been observed using a variety of methods ranging from fracture-flip replica staining, SEM as well as fluorescent super-resolution microscopy (Chasen et al., 2019; Pimenta et al., 1989; Vatarunakamura et al., 2005). There was also a notable compositional difference in the cytoskeleton directly beneath the POR membrane (Figure 4E arrowheads), inferring a likely influence on the PM domain above it. So, despite an obvious dearth in TM domain-containing proteins in this area, there is a clear enrichment of mannose-rich glycans suggesting that *whatever* is present on the POR surface, it is highly decorated with carbohydrate moieties. The role of these carbohydrate modifications in SPC function has not been conclusively determined, although reports suggest that they also play a role in endocytic activity (Brosson et al., 2016). Apart from its protein and carbohydrate composition, fracture-flip-based EM has also highlighted the POR surface membrane as both thicker and more roughly textured than the surrounding PM (Figure 4F; Pimenta et al., 1989). This difference in thickness is intriguing because the presence of sterols, e.g. ergosterol and cholesterol, is known to increase the observed width of membranes and this is especially true for ordered membrane domains such as lipid rafts (Yang et al., 2016). An important role for these membrane sterols in endocytosis is also supported by the observation that agents which bind to or deplete cholesterol, such as filipin or methyl-β-cyclodextrin (MβCD), completely inhibit cargo uptake (Correa et al., 2008). The rough texture of the POR, mentioned previously, has also been seen multiple times with various techniques including the first SEM picture of this structure (Figure 3C; Vatarunakamura et al., 2005). This texture is thought to relate back to the inherent “stickiness” of the region with the “rugous” appearance being a byproduct of material having been bound to the POR. Taken together, the SPC has been suggested to operate as a “sticky”, cholesterol-rich, detergent-resistant membrane with many of the hallmarks of a lipid raft domain (Correa et al., 2007). As we initiate a search for potential cargo receptors, it is reasonable to leverage these prior observations in order to establish a set of criteria for potential targets. The first and most obvious type of candidate receptor would be a glycosylphosphatidylinositol-anchored protein (GPI-AP). This is an attractive class of potential molecules for a number of reasons; first, we already know that the *T. brucei* transferrin receptor is a GPI-AP and so some precedent already exists in this parasite family even though no homologs of the ESAG6/7 receptor exists in the *T. cruzi* genome (Kariuki et al., 2019) and, second, it is the fact that GPI-APs are known to cluster in cholesterol-rich lipid raft domains similar to what we see in the POR. The POR itself could, therefore, function as an organizing platform in order to aggregate this class of membrane receptor (Denny et al., 2001; Edidin, 2003). The enforced clustering of GPI-APs in this region could also explain the preferential binding of ConA lectins to the POR which may be labeling the mannose-rich portion of the GPI anchor itself (Paulick & Bertozzi, 2008). However, it is worth noting that the ubiquitous labeling of other GPI-APs on the surface of *T. cruzi* in all life cycle stages makes it conceptually difficult to reason how partitioning of different types of GPI-APs at the PM would actually be accomplished in practice (Borges et al., 2021; Buscaglia et al., 2006; Freire-de-Lima et al., 2012). The freeze-fracture observations highlighting the low abundance of TM domain-containing proteins in the POR do not discount a potential role for this type of protein acting as a cargo receptor either. It has been observed that lipid rafts often have specific TM domain proteins associated with them, yet how these proteins selectively associate with lipid-ordered regions has itself been the subject of intense study. Numerous reports have shown that retention within lipid rafts often relies on a simple physical organizing principle; thicker lipid raft membranes can accommodate longer hydrophobic TM domains while simultaneously repelling shorter ones (Sharpe et al., 2010). Through a combination of biochemical techniques to enrich for detergent-resistant membranes, followed by selective enrichment of POR components using ConA pull-downs and mass spectrometry, it should be possible to begin identifying, in an unbiased way, potential receptor proteins associated with these areas of the PM. A bioinformatic search as well for proteins conserved in all SPC-containing organisms which retain; lectin domains, long (≥24 amino acids) hydrophobic TM domains and high expression in the replicative (i.e. active SPC) stages could provide additional criteria to identify cargo receptors from these analyses. In thinking about the capture of food to be endocytosed by this endocytic structure, it is enticing to imagine that this POR region may function as a lipid-raft platform of GPI-anchored and/or long TM domain receptor-like proteins that bind and signal internally to activate the endocytic machinery facilitating cargo uptake. Although there have been, as of yet, no cell surface cargo receptors characterized in any SPC-containing heterotroph, indirect observations support their existence (Alcantara et al., 2017) and the identification of the first feeding cargo receptor in *T. cruzi* will, no doubt, be an important step in understanding how this organism directly interacts with its host to acquire necessary nutrients.

## HOW IS CARGO PROPELLED DOWN THE SPC? SPECULATIONS ON THE IDENTITY AND ORGANIZATION OF SPC MACHINERY

Much has been made of the structure of the SPC in this review, but how does it actually work? At the moment we clearly do not have a full answer to this question, however, a good place to start may be in proposing a potential model that can serve as a tool to develop testable hypotheses. In this section, we will take the limited experimental data we currently have in order to propose a functional model for the SPC and highlight both important hints from the data and major gaps in our understanding that the field can now address. First, however, it is important that we briefly revisit findings from our recently published work on cytostomal myosins in order to make the case that a full molecular dissection of the SPC is indeed possible (Chasen et al., 2020).

As part of our analysis of the cytopharynx-targeted myosin motor MyoF, we generated parasite lines overexpressing a dominant-negative “rigor” mutant of this enzyme that, due to an inability to hydrolyze ATP and complete its power stroke, is unable to let go of actin filaments. Importantly, the overexpression of this rigor mutant made parasites completely defective in measurable endocytosis, suggesting that the inactive MyoF enzyme had poisoned the myosin motor network, thus stopping this process entirely. What was striking to us was that this endocytic block was not only *not* lethal, it had no significant effect at all on parasite growth or viability in *in vitro* culture. Following this observation, we generated a full deletion of the MyoF gene and, while not leading to an endocytic-null phenotype, reduced the endocytic rate by 86%. This *ΔMyoF* mutant again showed no measurable decrease in parasite growth or viability. We do not know how these parasites are able to survive in the absence of endocytosis but, as stated in the introduction, it may be due to surface membrane transporters that import simple nutrients that are provided in excess in the standard epimastigote liver infusion tryptose (LIT) media (Costa et al., 2020). This ability to make endocytic-null mutants, it turns out, may be extremely fortuitous, as the lack of a conditional knockdown system in *T. cruzi* continues to make the study of essential gene function exceedingly difficult (Chiurillo & Lander, 2021). This, therefore, opens up the possibility of using CRISPR/Cas9 gene-editing tools to begin ablating SPC components to directly assess their role in the endocytic process without compromising parasite fitness (Lander et al., 2017, 2019; Peng et al., 2014). While we have yet to thoroughly examine how the lack of endocytosis affects *in vivo* infection of *T. cruzi’s* vertebrate and invertebrate hosts, it is difficult to fathom that such a sophisticated system would have been retained across such long stretches of evolutionary time if it did not provide a clear benefit for the organism. We simply have not yet determined when, or under what conditions, the endocytic process indeed becomes essential. Importantly, this newfound capacity to interrogate SPC function has the unique potential of converting *T. cruzi* into a valuable model to study protozoan phagotrophy about which we also know surprisingly little.

**FIGURE 5:**
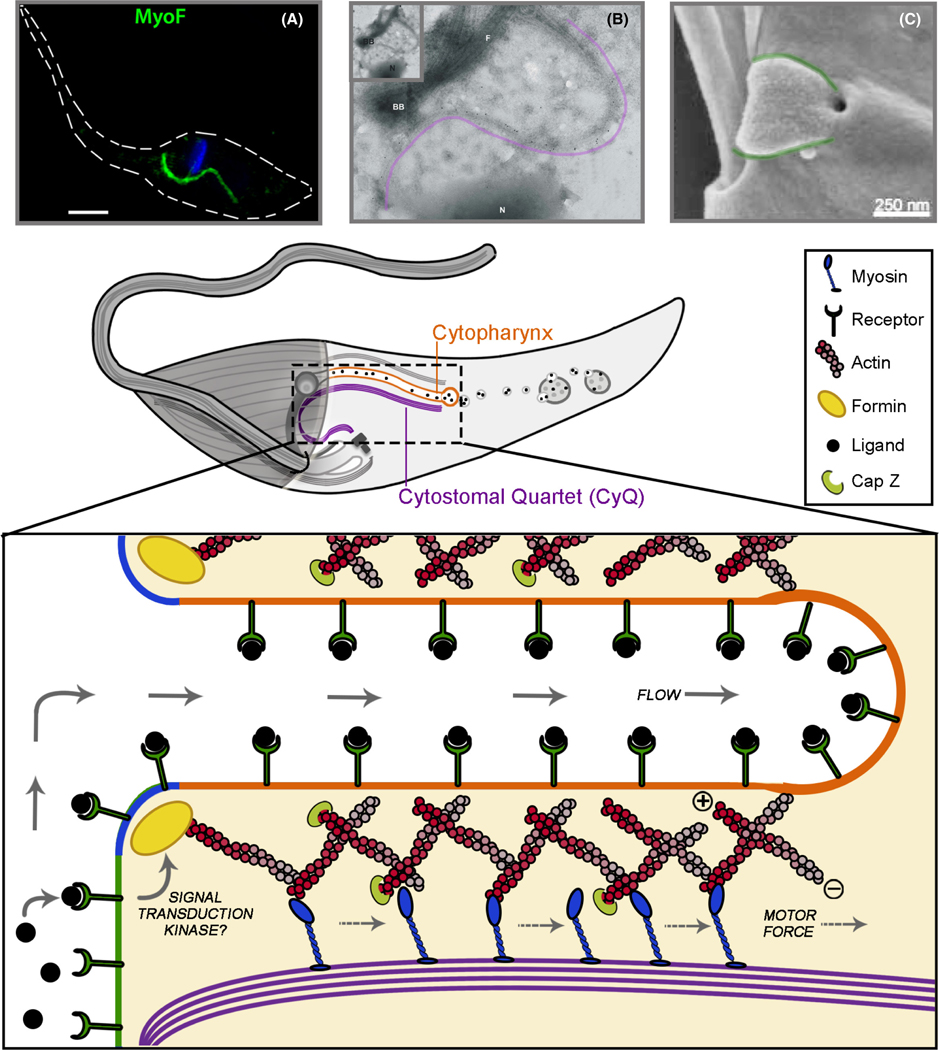
Proposed model for *T. cruzi* SPC-mediated endocytosis. Bottom: model for surface binding of endocytosed ligand to the surface receptor, followed by signal transduction to activate actin polymerization at SPC entrance and movement of actin coated membrane inward by CyQ/CyT anchored myosin motors. Location of actin regulating plus-end polymerase (yellow) and capping proteins (green) are noted. (A) Localization of MyoF (green) in *T. cruzi* epimastigotes. Adapted from Chasen et al. (2020). (B) Whole-mount immuno-EM with MyoF seen along SPC microtubules (purple line). Adapted from Alves et al. (2022). C, SEM of *T. cruzi* overexpressing TcVps34 highlighting altered POR prior to cytostome entrance (outlined in green). Adapted from Schoijet et al. (2008)

To begin constructing a potential model for SPC function, we should start with the first enzymatic component characterized; MyoF. Our initial localization of this molecular motor, using super-resolution microscopy, revealed MyoF to follow the corkscrew path of the SPC but, unfortunately, this technique did not allow us to see *exactly* where this protein was binding (Figure 5A). Likely scenarios involve either myosin tail attachment to cargo-laden vesicles and motor head movement along stable actin tracks or, conversely, tail binding to the SPC microtubule rootlets and the inward pulling of actin coated vesicles. Tail-based attachment to the microtubules was a favored model as we were able to show that the tail portions of these myosins were sufficient to send the fluorescent protein mNeon to the SPC structure, hence negating the need for the actin-binding motor head for SPC targeting. Cytochalasin treatment also appears to have no effect on MyoF localization at the SPC, again suggesting its association is actin independent. Direct evidence, however, has recently come from a follow-up study of MyoF by Alves et al. who, using immuno-EM labeling of tagged MyoF on whole-mount cytoskeletons of *T. cruzi*, showed that this motor protein directly associates with the rootlet microtubules (likely the CyQ although unclear) of the SPC (Figure 5B purple line; Alves et al., 2022). This observation lends credence to the idea that MyoF may, in fact, be fixed in place on the rootlets and act as a sort of “conveyer belt” for actin-coated endocytosed membrane. This might also explain how flooding the system with rigor mutants of MyoF could have impeded endocytosis with interspersed mutant enzymes irreversibly binding to and halting the inward flow of the endocytosed membrane. With MyoF fixed at the CyQ/CyT, the question then returns to the actin substrate of the SPC. The ability to visualize actin filaments has been extremely difficult in *T. cruzi* whether using EM or immunofluorescence microscopy (Souza et al., 1983). The majority of what we do know has come from a number of well-executed experimental studies and bioinformatic analyses of actin proteins in trypanosomatids which have shown that there are four different actin isoforms in *T. cruzi* while the related *T. brucei* has only a single isoform (Cevallos et al., 2011; Gupta et al., 2020; Vizcaino-Castillo et al., 2020; Vizcaíno-Castillo et al., 2019). A phylogenetic analysis revealed that, as compared to free-living *B. saltans*, *P. confusum* or *Leishmania* spp., isoforms 2 and 3 are consistently present in the genome of SPC-containing kinetoplastids. As a result, these seem like promising candidates although prior localization studies of Act2 did not show any clear localization to structures resembling the SPC within *T. cruzi* (Vizcaíno-Castillo et al., 2019). This could be for a variety of reasons including the short length or unbundled nature of actin microfilaments making visualization relative to the monomeric pools difficult. Since numerous studies using cytochalasin inhibitors of actin polymerization have shown this molecule to be essential for endocytosis, the identification of the actin isoform responsible will be an important step in constructing a likely model (Bogitsh et al., 1995; Chasen et al., 2019; Correa et al., 2008).

In thinking about the role of actin in the SPC, it should be reiterated that actin polymers are polar filaments with one end being referred to as barbed or plus (+) and the opposite end as pointed or minus (−; Figure 5 central schematic; Dominguez & Holmes, 2011). Polymerization of these filaments preferentially occurs at the plus end, while disassembly happens primarily at the minus end, resulting in a “treadmilling” effect that can be used to power a wide variety of cellular processes (Theriot, 2000). This filament polarity also facilitates molecular motors, such as myosins, moving in a single direction along the filament (Hartman & Spudich, 2012). Although the directionality of the myosin motors targeted to the SPC has not been experimentally determined, they are likely plus-end directed myosins based on structure and the extremely small number of minus-end directed myosins (type VI) which have been identified. With this constraint in mind, we can presume a necessity for the polarity of the actin network of the SPC in order to achieve the unidirectional flow of endocytosed membrane. With a fixed microtubule platform decorated with myosins in place (Figure 5 schematic), inward pulling of the cytopharynx membrane would require the plus ends (dark red) of the microfilament network coating the membrane to be oriented towards the cytostome opening and the minus end (light red) aiming toward the interior of the parasite. This orientation would, in many ways, be analogous to the actin network thought to support endocytosis in other vesicle trafficking systems as well (Kaksonen et al., 2006). Since, as stated previously, actin filaments in cells polymerize preferentially at the plus end and disassemble at the minus end, regulation of the location and timing of actin polymerization is likely an important point of control in the SPC-mediated endocytic process as well. One potential way of achieving this setup would be to target plus-end polymerizing enzymes to the SPC opening itself. In a recent bioinformatics analysis of actin and actin-regulating proteins, it was shown that *all* the human-infecting trypanosomatids have the ARP2/3 actin nucleating complex which supports branched actin networks as well as two plus-end polymerizing formin domain-containing proteins. Uniquely, however, *T. cruzi* and all other SPC-containing kinetoplastids retain a single additional formin isoform (TcCLB.511393.30) making it a promising candidate for this role (Gupta et al., 2020; Vizcaino-Castillo et al., 2020). This same analysis also noted that *T. brucei* and *Leishmania* spp. had lost both components (α and β subunits) of the plus-end capping complex CapZ. These capping proteins have the potential to regulate filament length or stability and therefore impact SPC function as well.

There are clearly a host of other components which are open to speculation regarding SPC function including the identity of the vesicular/tubular coat proteins, Rab GTPases and associated GAPs and GEFs, and various kinases or phosphatases that could regulate this process. With so many gaps in our understanding of SPC function, a good place to start might be to focus on the limited data we have regarding signaling events that initiate endocytosis in the first place. We can reasonably assume that once a ligand is bound to its receptor at the surface of *T. cruzi*, there would be a need to communicate this information across the plasma membrane to activate signaling cascades and initiate endocytosis. Prior work in *T. cruzi* has shown that the use of chemical inhibitors of both protein kinases and phosphatidylinositol-3 (PI3) kinases significantly diminish the endocytic rate and even cause overt surface membrane changes in the POR structure of parasites (Figure 5C; Correa et al., 2008; Schoijet et al., 2008). Kinases, it seems, could be central to this process through either modulating the activity of protein components directly or impacting the phosphatidylinositol phosphate makeup of the inner membrane which often serve as recruitment platforms for endocytic machinery (Wallroth & Haucke, 2018). It is worth pointing out that the details of how this, or any signaling pathway for that matter, operates in protozoans to sense the extracellular environment remain generally poorly understood (Subramanya & Mensa-Wilmot, 2010). This is due, in part, to the fact that standard mammalian signaling components are highly divergent or not present at all in these organisms. Kinetoplastids, for example, lack G-protein-coupled receptors entirely, have no equivalents of class I adenylyl cyclases, and no receptor ligands, agonists or antagonists have yet been identified (Gould & Koning, 2011). Even for the well-studied process of transferrin endocytosis in *T. brucei*, the full identity of signaling components involved remain a mystery, although the data clearly point to a role for kinases in this process as well (Horejsi et al., 1999; Joshi et al., 2007; Kariuki et al., 2019). Additionally, classical signaling is often carried out by tyrosine kinase modifications, yet the kinome of kinetoplastids is devoid of tyrosine kinase and tyrosine kinase-like groups (Bahia et al., 2009; Parsons et al., 2005). Despite this, the phospho-proteome of *T. cruzi* has revealed the presence of phosphorylated tyrosine, suggesting that this modification is, most likely, being carried out by either atypical tyrosine kinases or dual-specificity kinases that can phosphorylate serine, threonine, and tyrosine (Amorim et al., 2017; Marchini et al., 2011). The identity of a true tyrosine kinase in *T. cruzi*, however, remains to be demonstrated. The previously mentioned analysis of the kinetoplastid kinome has also revealed the existence of several putative kinases, either present only in *T. cruzi* or SPC-containing kinetoplastids that are promising targets for gene deletion studies that could be an important step forward in dissecting signaling networks regulating endocytosis. There are, no doubt, additional dimensions of this process that merit speculation and we suspect that there is a high probability that many of the hypotheses we propose here will not stand up to empirical testing. Only through experimentation and refinement of this model, we will arrive at a topological and mechanistic understanding of the molecular machinery driving this process.

## CONCLUDING REMARKS

We would like to end this review by noting that until now, we have focused primarily on the basic mechanics of the SPC and how it might operate. This fixation on the “how” of the SPC in the end fails to address an equally important and nevertheless often neglected “why” question; *Why were the salivarians able to abandon the SPC while the majority of trypanosomatids were compelled to retain it? What exactly can a cytostome-cytopharynx do that a flagellar pocket cannot?* In thinking about other intestinal parasites which are fecal-oral transmitted, the monoxenous apicomplexans, for example, one quickly realizes that these organisms interact with their hosts in a fundamentally different way from the trypanosomatids. The apicomplexans do not rely on food ingested by their host for sustenance, instead they employ a type of sophisticated myzocytosis or “cellular vampirism” that directly extracts cytosolic nutrients from the host’s intestinal cells (Gubbels & Duraisingh, 2012). The trypanosomatids, on the other hand, operate more like members of the arthropod microbiome as they never directly target or consume their host’s tissues (Schaub, 2021). Assuming that ancestral kinetoplastids were likely free-living and consumed bacteria, it is tempting to speculate that the early association between these proto-parasites and the intestinal milieu of arthropods may have been originally based on the consumption of the resident microflora itself. This mirrors what can be seen today with our own resident protozoan excavate *Chilomastix mesnili* which is nonpathogenic and feeds on our intestinal bacteria (Zaman et al., 2000). The establishment of a replicative niche could have then allowed the subsequent diversification of feeding targets to also include food ingested by the bug itself, thus opening the door to these kinetoplastids abandoning bacterial predation entirely. This change in food source would have, in turn, promoted a reliance on the vector’s own eating habits, thus setting them in direct competition for nutrients and space not only with their host’s digestive system but also with other bacterial or fungal constituents of the microbiome. Is it possible then to consider that trypanosomatids may never have fully given up this ability to phagocytose microbes? Could a reversion back to an ancestral state of consuming prokaryotic prey be a potential mechanism to explain how *T. cruzi* is able to survive such long periods of time between vector blood meals (Kollien & Schaub, 1998)? This is clearly an unorthodox proposition that lacks any supporting data, but it would, in the end, be one of the simplest hypotheses for why this expansive group of organisms kept this complex structure. This is an intriguing premise and with the ability to generate endocytic-null mutants, we can now begin directly testing what effect a loss of endocytosis may have on *T. cruzi’s* ability to colonize its insect vector.

In conclusion, our overarching goal with this review has been simply to show both how much progress the field has made in dissecting the structure of the *T. cruzi* endocytic organelle and also illustrate how much further we still have to go to fully understand this process. Using the many seminal contributions which have shaped our perception of the SPC, we have ended this review by presenting a testable model with which to begin interrogating how this oral apparatus both captures, activates, and pulls material in. We believe that the continued investigation into this fascinatingly complex oral apparatus has the potential to not only impact our ability to combat agents of human disease, but also shed light on the evolutionary past and ecological presence of these important phagotrophic protozoans. With our recently published studies of the SPC myosin motors, we have only just begun to decipher how it is that a single-celled protozoan is able to spatially and temporally construct and control such a diverse array of components in order to engage in this unique mode of endocytosis. By combining the wealth of genomic and structural data already in hand, with the recent advances in available molecular tools to manipulate the genome of *T. cruzi*, the field is well positioned to begin elucidating the mechanistic basis of this ancient protozoal feeding apparatus with the goal of providing insight into fundamental processes ranging from global microbial food webs to parasitic diseases.

## Figures and Tables

**FIGURE 1:**
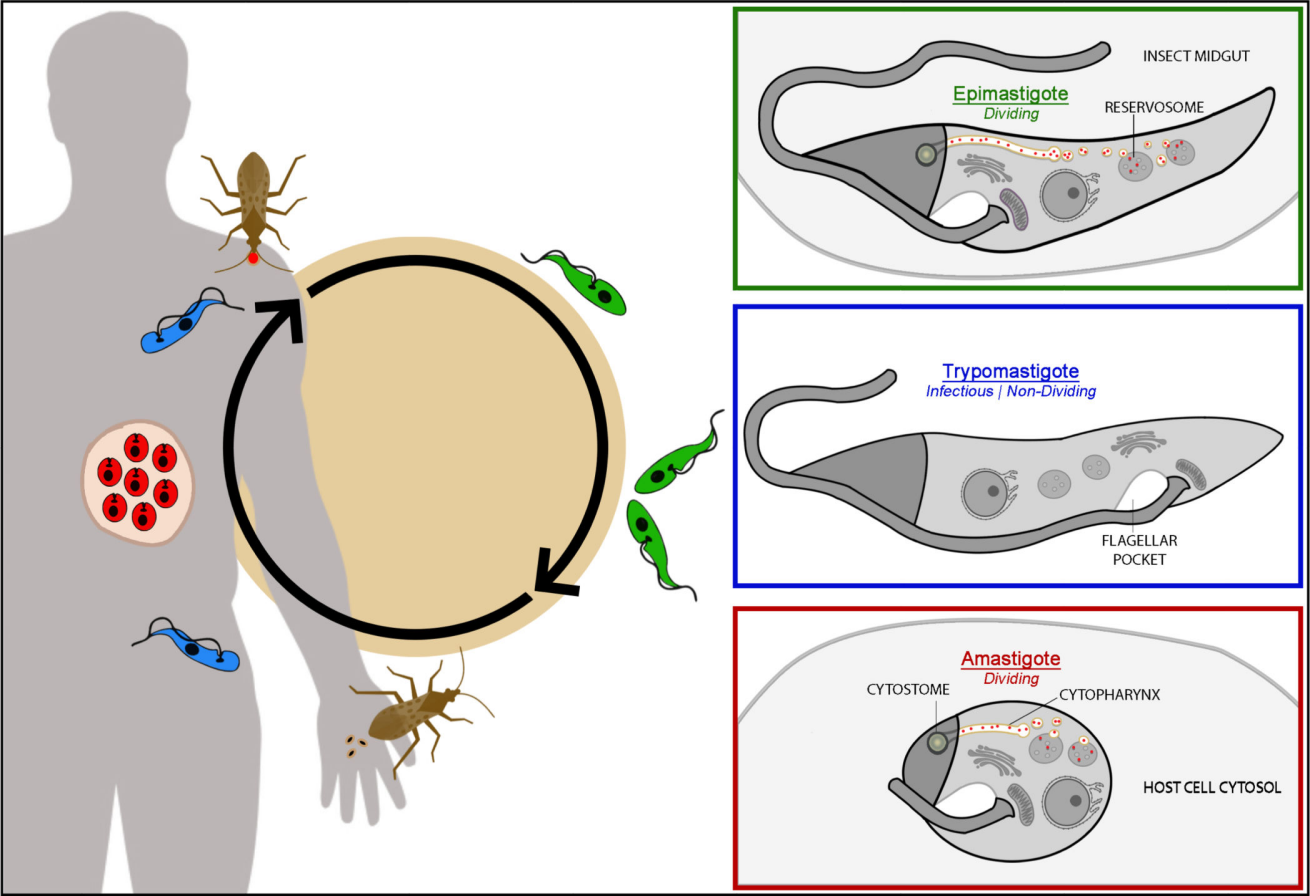
Life cycle of *Trypanosoma cruzi* and dynamics of the cytostome-cytopharynx (SPC) endocytic organelle. Actively dividing epimastigotes (green) endocytose via the SPC and colonize the insect vector gastrointestinal tract. In the vector hindgut, parasites transform into metacyclic trypomastigotes (blue) break down the SPC and are excreted onto mammalian hosts. These infectious and nondividing forms of the parasite invade host cells and replicate cytosolically as amastigotes (red). The amastigotes phagocytose via the SPC before regenerating their flagellum and converting into infectious trypomastigotes (blue). These forms are able to reinvade additional host cells or are taken up by the vector during a blood meal to complete the life cycle

## References

[R1] Adl SM, Simpson AG, Lane CE, Lukes J, Bass D, Bowser SS (2012) The revised classification of eukaryotes. Journal of Eukaryotic Microbiology, 59(5), 429–493. 10.1111/j.1550-7408.2012.00644.x 23020233 PMC3483872

[R2] Alcantara CL, de Souza W. & da Cunha ESNL (2018) Tridimensional electron microscopy analysis of the early endosomes and endocytic traffic in *Trypanosoma cruzi* epimastigotes. Protist, 169(6), 887–910. 10.1016/j.protis.2018.09.004 30447618

[R3] Alcantara CL, de Souza W. & Cunha ESNL (2021) The cytostome-cytopharynx complex of intracellular and extracellular amastigotes of *Trypanosoma cruzi* exhibit structural and functional differences. Cellular Microbiology, 23(9), e13346. 10.1111/cmi.13346 33900003

[R4] Alcantara CL, Vidal JC, de Souza W. & Cunha-e-Silva NL (2014) The three-dimensional structure of the cytostome-cytopharynx complex of *Trypanosoma cruzi* epimastigotes. Journal of Cell Science, 127(Pt 10), 2227–2237. 10.1242/jcs.135491 24610945

[R5] Alcantara CL, Vidal JC, de Souza W. & Cunha ESNL (2017) The cytostome-cytopharynx complex of *Trypanosoma cruzi* epimastigotes disassembles during cell division. Journal of Cell Science, 130(1), 164–176. 10.1242/jcs.187419 27363990

[R6] Alves AA, Alcantara CL, Dantas-Jr MVA, Sunter JD, De Souza W. & Cunha ESNL (2022) Dynamics of the orphan myosin MyoF over *Trypanosoma cruzi* life cycle and along the endocytic pathway. Parasitology International, 86, 102444. 10.1016/j.parint.2021.102444 34464754

[R7] Amorim JC, Batista M, da Cunha ES, Lucena ACR, Lima CVP, Sousa K. (2017) Quantitative proteome and phosphoproteome analyses highlight the adherent population during *Trypanosoma cruzi* metacyclogenesis. Scientific Reports, 7(1), 9899. 10.1038/s41598-017-10292-3 28852088 PMC5574995

[R8] Bahia D, Oliveira LM, Lima FM, Oliveira P, Silveira JF, Mortara RA (2009) The TryPIKinome of five human pathogenic trypanosomatids: *Trypanosoma brucei*, *Trypanosoma cruzi*, *Leishmania major*, *Leishmania braziliensis* and *Leishmania infantum*— new tools for designing specific inhibitors. Biochemical and Biophysical Research Communications, 390(3), 963–970. 10.1016/j.bbrc.2009.10.086 19852933

[R9] Barrett MP, Burchmore RJ, Stich A, Lazzari JO, Frasch AC, Cazzulo JJ (2003) The trypanosomiases. The Lancet, 362(9394), 1469–1480. 10.1016/S0140-6736(03)14694-6 14602444

[R10] Boeck WC (1921) Chilomastix mesnili and a method for its culture. Journal of Experimental Medicine, 33(2), 147–175. 10.1084/jem.33.2.147 19868485 PMC2128178

[R11] Bogitsh BJ, Ribeiro-Rodrigues R. & Carter CE (1995) In vitro effects of mannan and cytochalasin B on the uptake of horseradish peroxidase and [14C]sucrose by *Trypanosoma cruzi* epimastigotes. Journal of Parasitology, 81(2), 144–148. 7707187

[R12] Borges AR, Link F, Engstler M. & Jones NG (2021) The glycosylphosphatidylinositol anchor: a linchpin for cell surface versatility of trypanosomatids. *Frontiers in Cell and* Developmental Biology, 9, 720536. 10.3389/fcell.2021.720536 PMC8591177 34790656

[R13] Bretana A. & O’Daly JA (1976) Uptake of fetal proteins by *Trypanosoma cruzi* immunofluorescence and ultrastructural studies. International Journal for Parasitology, 6(5), 379–386. 786918 10.1016/0020-7519(76)90022-9

[R14] Brosson S, Bottu G, Pays E, Bousbata S. & Salmon D. (2017) Identification and preliminary characterization of a putative C-type lectin receptor-like protein in the *T. cruzi* tomato lectin endocytic-enriched proteome. Microbiological Research, 205, 73–79. 10.1016/j.micres.2017.07.001 28942847

[R15] Brosson S, Fontaine F, Vermeersch M, Perez-Morga D, Pays E, Bousbata S. (2016) Specific endocytosis blockade of *Trypanosoma cruzi* exposed to a poly-LAcNAc binding lectin suggests that lectin-sugar interactions participate to receptor-mediated endocytosis. PLoS One, 11(9), e0163302. 10.1371/journal.pone.0163302 PMC5042520 27685262

[R16] Burza S, Croft SL & Boelaert M. (2018) Leishmaniasis. The Lancet, 392(10151), 951–970. 10.1016/S0140-6736(18)31204-2 30126638

[R17] Buscaglia CA, Campo VA, Frasch AC & Di Noia JM (2006) *Trypanosoma cruzi* surface mucins: host-dependent coat diversity. Nature Reviews Microbiology, 4(3), 229–236. 10.1038/nrmicro1351 16489349

[R18] Buscher P, Cecchi G, Jamonneau V. & Priotto G. (2017) Human African trypanosomiasis. The Lancet, 390(10110), 2397–2409. 10.1016/S0140-6736(17)31510-6 28673422

[R19] Cavalier-Smith T. (2002) The phagotrophic origin of eukaryotes and phylogenetic classification of Protozoa. International Journal of Systematic and Evolutionary Microbiology, 52(Pt 2), 297–354. 10.1099/00207713-52-2-297 11931142

[R20] Cevallos AM, Segura-Kato YX, Merchant-Larios H, Manning-Cela R, Alberto Hernandez-Osorio L, Marquez-Duenas C. (2011) *Trypanosoma cruzi*: multiple actin isovariants are observed along different developmental stages. Experimental Parasitology, 127(1), 249–259. 10.1016/j.exppara.2010.08.003 20705070

[R21] Chasen NM, Coppens I. & Etheridge RD (2019) Identification and localization of the first known proteins of the *Trypanosoma cruzi* Cytostome Cytopharynx endocytic complex. Frontiers in Cellular and Infection Microbiology, 9, 445. 10.3389/fcimb.2019.00445 32010635 PMC6978632

[R22] Chasen NM, Etheridge MG & Etheridge RD (2020) The functional characterization of TcMyoF implicates a family of cytostome-cytopharynx targeted myosins as integral to the endocytic machinery of *Trypanosoma cruzi*. mSphere, 5(3). 10.1128/mSphere.00313-20 PMC7300353 32554712

[R23] Chiari E, de Souza W, Romanha AJ, Chiari CA & Brener Z. (1978) Concanavalin a receptors on the cell membrane of *Trypanosoma cruzi*. Acta Tropica, 35(2), 113–121. 28652

[R24] Chiurillo MA & Lander N. (2021) The long and winding road of reverse genetics in *Trypanosoma cruzi*. Microbial Cell, 8(9), 203–207. 10.15698/mic2021.09.758 34527719 PMC8404153

[R25] Corliss JO (1959) An illustrated key to the higher groups of the ciliated protozoa, with definition of terms. Journal of Protozoology, 6(3), 265–281. 10.1111/j.1550-7408.1959.tb04368.x

[R26] Correa JR, Atella GC, Vargas C. & Soares MJ (2007) Transferrin uptake may occur through detergent-resistant membrane domains at the cytopharynx of *Trypanosoma cruzi* epimastigote forms. Memorias do Instituto Oswaldo Cruz, 102(7), 871–876. 17992361 10.1590/s0074-02762007005000117

[R27] Correa JR, Atella GC, Batista MM & Soares MJ (2008) Transferrin uptake in *Trypanosoma cruzi* is impaired by interference on cytostome-associated cytoskeleton elements and stability of membrane cholesterol, but not by obstruction of clathrin-dependent endocytosis. Experimental Parasitology, 119(1), 58–66. 10.1016/j.exppara.2007.12.010 18234197

[R28] Costa AFP, de Brito RCF, Carvalho LM, Cardoso JMO, Vieira PMA, Reis AB (2020) Liver infusion tryptose (LIT): the best choice for growth, viability, and infectivity of *Leishmania infantum* parasites. Parasitology Research, 119(12), 4185–4195. 10.1007/s00436-020-06893-z 33033848 PMC7544523

[R29] De Souza W. (2002) Basic cell biology of *Trypanosoma cruzi*. Current Pharmaceutical Design, 8(4), 269–285. 11860366 10.2174/1381612023396276

[R30] De Souza W. & Barrias ES (2020) May the epimastigote form of *Trypanosoma cruzi* be infective? Acta Tropica, 212, 105688. 10.1016/j.actatropica.2020.105688 32888934

[R31] De Souza W, Martinez-Palomo A. & Gonzalez-Robles A. (1978) The cell surface of *Trypanosoma cruzi*: cytochemistry and freeze-fracture. Journal of Cell Science, 33, 285–299. 363731 10.1242/jcs.33.1.285

[R32] Denny PW, Field MC & Smith DF (2001) GPI-anchored proteins and glycoconjugates segregate into lipid rafts in Kinetoplastida. FEBS Letters, 491(1– 2), 148–153. 11226438 10.1016/s0014-5793(01)02172-x

[R33] Deschamps P, Lara E, Marande W, Lopez-Garcia P, Ekelund F. & Moreira D. (2011) Phylogenomic analysis of kinetoplastids supports that trypanosomatids arose from within bodonids. Molecular Biology and Evolution, 28(1), 53–58. 10.1093/molbev/msq289 21030427

[R34] Dominguez R. & Holmes KC (2011) Actin structure and function. Annual Review of Biophysics, 40, 169–186. 10.1146/annurev-biophys-042910-155359 PMC3130349 21314430

[R35] Dong X, Lim TK, Lin Q. & He CY (2020) Basal body protein TbSAF1 is required for microtubule quartet anchorage to the basal bodies in *Trypanosoma brucei*. MBio, 11(3). 10.1128/mBio.00668-20 PMC7291619 32518185

[R36] Edidin M. (2003) The state of lipid rafts: from model membranes to cells. Annual Review of Biophysics and Biomolecular Structure, 32, 257–283. 10.1146/annurev.biophys.32.110601.142439 12543707

[R37] Field MC & Carrington M. (2009) The trypanosome flagellar pocket. Nature Reviews Microbiology, 7(11), 775–786. 10.1038/nrmicro2221 19806154

[R38] Flegontov P, Votypka J, Skalicky T, Logacheva MD, Penin AA, Tanifuji G. (2013) Paratrypanosoma is a novel early-branching trypanosomatid. Current Biology, 23(18), 1787–1793. 10.1016/j.cub.2013.07.045 24012313

[R39] Flegontova O, Flegontov P, Malviya S, Poulain J, de Vargas C, Bowler C. (2018) Neobodonids are dominant kinetoplastids in the global ocean. Environmental Microbiology, 20(2), 878–889. 10.1111/1462-2920.14034 29266706

[R40] Freire-de-Lima L, Oliveira IA, Neves JL, Penha LL, Alisson-Silva F, Dias WB (2012) Sialic acid: a sweet swing between mammalian host and *Trypanosoma cruzi*. Frontiers in Immunology, 3, 356. 10.3389/fimmu.2012.00356 23230438 PMC3515882

[R41] Girard-Dias W, Alcantara CL, Cunha-e-Silva N, de Souza W. & Miranda K. (2012) On the ultrastructural organization of *Trypanosoma cruzi* using cryopreparation methods and electron tomography. Histochemistry and Cell Biology, 138(6), 821–831. 10.1007/s00418-012-1002-8 22872316

[R42] Gould MK & de Koning HP (2011) Cyclic-nucleotide signalling in protozoa. FEMS Microbiology Reviews, 35(3), 515–541. 10.1111/j.1574-6976.2010.00262.x 21223322

[R43] Groom ZC, Protopapas AD & Zochios V. (2017) Tropical diseases of the myocardium: a review. International Journal of General Medicine, 10, 101–111. 10.2147/IJGM.S130828 28435310 PMC5391162

[R44] Gubbels MJ & Duraisingh MT (2012) Evolution of apicomplexan secretory organelles. International Journal for Parasitology, 42(12), 1071–1081. 10.1016/j.ijpara.2012.09.009 23068912 PMC3583008

[R45] Gupta CM, Ambaru B. & Bajaj R. (2020) Emerging functions of actins and actin binding proteins in trypanosomatids. Frontiers in Cell and Developmental Biology, 8, 587685. 10.3389/fcell.2020.587685 PMC7581878 33163497

[R46] Harmer J, Yurchenko V, Nenarokova A, Lukes J. & Ginger ML (2018) Farming, slaving and enslavement: histories of endosymbioses during kinetoplastid evolution. Parasitology, 145, 1311–1323. 10.1017/S0031182018000781 29895336

[R47] Harp JA (2003) Parasitic infections of the gastrointestinal tract. Current Opinion in Gastroenterology, 19(1), 31–36. 15699890 10.1097/00001574-200301000-00005

[R48] Hartman MA & Spudich JA (2012) The myosin superfamily at a glance. Journal of Cell Science, 125(7), 1627–1632. 10.1242/jcs.094300 22566666 PMC3346823

[R49] Hoog JL, Lacomble S, Bouchet-Marquis C, Briggs L, Park K, Hoenger A. (2016) 3D architecture of the Trypanosoma brucei flagella connector, a mobile transmembrane junction. PLoS Neglected Tropical Diseases, 10(1), e0004312. 10.1371/journal.pntd.0004312 PMC4731218 26820516

[R50] Horejsi V, Drbal K, Cebecauer M, Cerny J, Brdicka T, Angelisova P. (1999) GPI-microdomains: a role in signalling via immunoreceptors. Immunology Today, 20(8), 356–361. 10.1016/S0167-5699(99)01489-9 10431155

[R51] Joshi MB, Ivanov D, Philippova M, Erne P. & Resink TJ (2007) Integrin-linked kinase is an essential mediator for T-cadherin-dependent signaling via Akt and GSK3beta in endothelial cells. The FASEB Journal, 21(12), 3083–3095. 10.1096/fj.06-7723com 17485554

[R52] Kaksonen M, Toret CP & Drubin DG (2006) Harnessing actin dynamics for clathrin-mediated endocytosis. Nature Reviews Molecular Cell Biology, 7(6), 404–414. 10.1038/nrm1940 16723976

[R53] Kariuki CK, Stijlemans B. & Magez S. (2019) The trypanosomal transferrin receptor of *Trypanosoma brucei*— a review. Tropical Medicine and Infectious Disease, 4(4), 126. 10.3390/tropicalmed4040126 31581506 PMC6958415

[R54] Kollien AH & Schaub GA (1998) The development of *Trypanosoma cruzi* (Trypanosomatidae) in the reduviid bug Triatoma infestans (Insecta): influence of starvation. Journal of Eukaryotic Microbiology, 45(1), 59–63. 9495034 10.1111/j.1550-7408.1998.tb05070.x

[R55] Lander N, Chiurillo MA, Vercesi AE & Docampo R. (2017) Endogenous C-terminal tagging by CRISPR/Cas9 in *Trypanosoma cruzi*. BIO-PROTOCOL, 7(10). 10.21769/BioProtoc.2299 PMC5531189 28758140

[R56] Lander N, Chiurillo MA & Docampo R. (2019) Genome editing by CRISPR/Cas9 in *Trypanosoma cruzi*. In: Gómez K. & Buscaglia C. (Eds.) T. cruzi infection. Methods in molecular biology NewYork, NY: Humana Press, vol. 1955. 10.1007/978-1-4939-9148-8_5 30868519

[R57] Landfear SM & Ignatushchenko M. (2001) The flagellum and flagellar pocket of trypanosomatids. Molecular and Biochemical Parasitology, 115(1), 1–17. 11377735 10.1016/s0166-6851(01)00262-6

[R58] Losinno AD, Martinez SJ, Labriola CA, Carrillo C. & Romano PS (2020) Induction of autophagy increases the proteolytic activity of reservosomes during *Trypanosoma cruzi* metacyclogenesis. Autophagy, 17(2), 439–456. 10.1080/15548627.2020.1720428 31983275 PMC8007142

[R59] Lukes J, Butenko A, Hashimi H, Maslov DA, Votypka J. & Yurchenko V. (2018) Trypanosomatids are much more than just trypanosomes: clues from the expanded family tree. Trends in Parasitology, 34(6), 466–480. 10.1016/j.pt.2018.03.002 29605546

[R60] Lukes J, Skalicky T, Tyc J, Votypka J. & Yurchenko V. (2014) Evolution of parasitism in kinetoplastid flagellates. Molecular and Biochemical Parasitology, 195(2), 115–122. 10.1016/j.molbiopara.2014.05.007 24893339

[R61] Manne-Goehler J, Umeh CA, Montgomery SP & Wirtz VJ (2016) Estimating the burden of Chagas disease in the United States. PLoS Neglected Tropical Diseases, 10(11), e0005033. 10.1371/journal.pntd.0005033 PMC5098725 27820837

[R62] Marchese L, Nascimento JF, Damasceno FS, Bringaud F, Michels PAM & Silber AM (2018) The uptake and metabolism of amino acids, and their unique role in the biology of pathogenic Trypanosomatids. Pathogens, 7(2), 36. 10.3390/pathogens7020036 29614775 PMC6027508

[R63] Marchini FK, de Godoy LM, Rampazzo RC, Pavoni DP, Probst CM, Gnad F. (2011) Profiling the *Trypanosoma cruzi* phosphoproteome. PLoS One, 6(9), e25381. 10.1371/journal.pone.0025381 PMC3178638 21966514

[R64] Martel CM (2009) Conceptual bases for prey biorecognition and feeding selectivity in the microplanktonic marine phagotroph Oxyrrhis marina. Microbial Ecology, 57(4), 589–597. 10.1007/s00248-008-9421-8 18642040

[R65] Martinez-Palomo A, DeSouza W. & Gonzalez-Robles A. (1976) Topographical differences in the distribution of surface coat components and intramembrane particles. A cytochemical and freeze-fracture study in culture forms of *Trypanosoma cruzi*. Journal of Cell Biology, 69(2), 507–513. 770483 10.1083/jcb.69.2.507 PMC2109674

[R66] Mathers CD, Lopez A. & Murray C. (2006) The burden of disease and mortality by condition: data, methods, and results for the year 2001. In: Lopez A, Ezzati M, Jamison D. & Murray C. (Eds.) Global burden of disease and risk factors. New York: Oxford University Press.

[R67] Meissner M, Agop-Nersesian C. & Sullivan WJ Jr. (2007) Molecular tools for analysis of gene function in parasitic microorganisms. Applied Microbiology and Biotechnology, 75(5), 963–975. 10.1007/s00253-007-0946-4 17401559

[R68] Meyer H. & de Souza W. (1973) On the fine structure of *Trypanosoma cruzi* in tissue cultures of pigment epithelium from the chick embryo. Uptake of melanin granules by the parasite. The Journal of Protozoology, 20(5), 590–593. 4128532 10.1111/j.1550-7408.1973.tb03580.x

[R69] Michels PAM, Villafraz O, Pineda E, Alencar MB, Caceres AJ, Silber AM (2021) Carbohydrate metabolism in trypanosomatids: new insights revealing novel complexity, diversity and species-unique features. Experimental Parasitology, 224, 108102. 10.1016/j.exppara.2021.108102 33775649

[R70] Milder R. & Deane MP (1969) The cytostome of *Trypanosoma cruzi* and *T. conorhini*. The Journal of Protozoology, 16(4), 730–737. 5362390 10.1111/j.1550-7408.1969.tb02335.x

[R71] Morgan GW, Hall BS, Denny PW, Carrington M. & Field MC (2002) The kinetoplastida endocytic apparatus. Part I: a dynamic system for nutrition and evasion of host defences. Trends in Parasitology, 18(11), 491–496. 12473365 10.1016/s1471-4922(02)02391-7

[R72] Morgan GW, Hall BS, Denny PW, Field MC & Carrington M. (2002) The endocytic apparatus of the kinetoplastida. Part II: machinery and components of the system. Trends in Parasitology, 18(12), 540–546. 12482539 10.1016/s1471-4922(02)02392-9

[R73] Nickerson P, Orr P, Schroeder ML, Sekla L. & Johnston JB (1989) Transfusion-associated *Trypanosoma cruzi* infection in a non-endemic area. Annals of Internal Medicine, 111(10), 851–853. 2510572 10.7326/0003-4819-111-10-851

[R74] Okuda K, Esteva M, Segura EL & Bijovsy AT (1999) The cytostome of *Trypanosoma cruzi* epimastigotes is associated with the flagellar complex. Experimental Parasitology, 92(4), 223–231. 10.1006/expr.1999.4419 10425150

[R75] Opperdoes FR, Butenko A, Flegontov P, Yurchenko V. & Lukes J. (2016) Comparative metabolism of free-living bodo saltans and parasitic trypanosomatids. Journal of Eukaryotic Microbiology, 63(5), 657–678. 10.1111/jeu.12315 27009761

[R76] Overath P. & Engstler M. (2004) Endocytosis, membrane recycling and sorting of GPI-anchored proteins: *Trypanosoma brucei* as a model system. Molecular Microbiology, 53(3), 735–744. 10.1111/j.1365-2958.2004.04224.x 15255888

[R77] Parsons M, Worthey EA, Ward PN & Mottram JC (2005) Comparative analysis of the kinomes of three pathogenic trypanosomatids: *Leishmania major*, *Trypanosoma brucei* and *Trypanosoma cruzi*. BMC Genomics, 6, 127. 10.1186/1471-2164-6-127 16164760 PMC1266030

[R78] Paulick MG & Bertozzi CR (2008) The glycosylphosphatidylinositol anchor: a complex membrane-anchoring structure for proteins. Biochemistry, 47(27), 6991–7000. 10.1021/bi8006324 18557633 PMC2663890

[R79] Pelosse P. & Kribs-Zaleta CM (2012) The role of the ratio of vector and host densities in the evolution of transmission modes in vector-borne diseases. The example of sylvatic *Trypanosoma cruzi*. Journal of Theoretical Biology, 312, 133–142. 10.1016/j.jtbi.2012.07.028 22892441

[R80] Peng D, Kurup SP, Yao PY, Minning TA & Tarleton RL (2014) CRISPR-Cas9-mediated single-gene and gene family disruption in *Trypanosoma cruzi*. MBio, 6(1), e02097–14. 10.1128/mBio.02097-14 PMC4281920 25550322

[R81] Perez-Molina JA & Molina I. (2018) Chagas disease. The Lancet, 391(10115), 82–94. 10.1016/S0140-6736(17)31612-4 28673423

[R82] Pimenta PF, de Souza W, Souto-Padron T. & Pinto da Silva P. (1989) The cell surface of *Trypanosoma cruzi*: a fracture-flip, replica-staining label-fracture survey. European Journal of Cell Biology, 50(2), 263–271. 2483376

[R83] Pitelka DR (1961) Observations on the kinetoplast-mitochondrion and the cytostome of Bodo. Experimental Cell Research, 25(1), 87–93. 10.1016/0014-4827(61)90309-3 14486970

[R84] Porto-Carreiro I, Attias M, Miranda K, De Souza W. & Cunha-e-Silva N. (2000) *Trypanosoma cruzi* epimastigote endocytic pathway: cargo enters the cytostome and passes through an early endosomal network before storage in reservosomes. European Journal of Cell Biology, 79(11), 858–869. 10.1078/0171-9335-00112 11139150

[R85] Preston TM (1969) The form and function of the cytostome-cytopharynx of the culture forms of the elasmobranch haemoflagellate *Trypanosoma raiae* Laveran & Mesnil. The Journal of Protozoology, 16(2), 320–333. 5796880 10.1111/j.1550-7408.1969.tb02278.x

[R86] Ramos TC, Freymuller-Haapalainen E. & Schenkman S. (2011) Three-dimensional reconstruction of *Trypanosoma cruzi* epimastigotes and organelle distribution along the cell division cycle. Cytometry A, 79(7), 538–544. 10.1002/cyto.a.21077 21567937

[R87] Ritchie K, Iino R, Fujiwara T, Murase K. & Kusumi A. (2003) The fence and picket structure of the plasma membrane of live cells as revealed by single molecule techniques (Review). Molecular Membrane Biology, 20(1), 13–18. 12745919 10.1080/0968768021000055698

[R88] Roberts EC, Zubkov MV, Martin-Cereceda M, Novarino G. & Wootton EC (2006) Cell surface lectin-binding glycoconjugates on marine planktonic protists. FEMS Microbiology Letters, 265(2), 202–207. 10.1111/j.1574-6968.2006.00484.x 17147765

[R89] Saha S, Anilkumar AA & Mayor S. (2016) GPI-anchored protein organization and dynamics at the cell surface. Journal of Lipid Research, 57(2), 159–175. 10.1194/jlr.R062885 26394904 PMC4727430

[R90] Schaub GA (2021) An update on the knowledge of parasite-vector interactions of chagas disease. Research and Reports in Tropical Medicine, 12, 63–76. 10.2147/RRTM.S274681 34093053 PMC8169816

[R91] Schoijet AC, Miranda K, Girard-Dias W, de Souza W, Flawia MM, Torres HN (2008) A *Trypanosoma cruzi* phosphatidylinositol 3-kinase (TcVps34) is involved in osmoregulation and receptor-mediated endocytosis. Journal of Biological Chemistry, 283(46), 31541–31550. 10.1074/jbc.M801367200 18801733 PMC2581564

[R92] Scott DA, Docampo R, Dvorak JA, Shi S. & Leapman RD (1997) In situ compositional analysis of acidocalcisomes in *Trypanosoma cruzi*. Journal of Biological Chemistry, 272(44), 28020–28029. 9346954 10.1074/jbc.272.44.28020

[R93] Sharpe HJ, Stevens TJ & Munro S. (2010) A comprehensive comparison of transmembrane domains reveals organelle-specific properties. Cell, 142(1), 158–169. 10.1016/j.cell.2010.05.037 20603021 PMC2928124

[R94] Sherr EB & Sherr BF (2002) Significance of predation by protists in aquatic microbial food webs. Antonie van Leeuwenhoek, 81(1– 4), 293–308. 10.1023/a:1020591307260 12448728

[R95] Simpson AG, Lukes J. & Roger AJ (2002) The evolutionary history of kinetoplastids and their kinetoplasts. Molecular Biology and Evolution, 19(12), 2071–2083. 10.1093/oxfordjournals.molbev.a004032 12446799

[R96] Sinha R, Rajesh A, Rawat S, Rajiah P. & Ramachandran I. (2012) Infections and infestations of the gastrointestinal tract. Part 2: parasitic and other infections. Clinical Radiology, 67(5), 495–504. 10.1016/j.crad.2011.10.022 22169349

[R97] Skalicky T, Dobakova E, Wheeler RJ, Tesarova M, Flegontov P, Jirsova D. (2017) Extensive flagellar remodeling during the complex life cycle of Paratrypanosoma, an early-branching trypanosomatid. Proceedings of the National Academy of Sciences of the United States of America, 114(44), 11757–11762. 10.1073/pnas.1712311114 29078369 PMC5676924

[R98] Soares MJ & De Souza W. (1988) Cytoplasmic organelles of trypanosomatids: a cytochemical and stereological study. Journal of Submicroscopic Cytology and Pathology, 20(2), 349–361. 3135113

[R99] Soares MJ, Souto-Padron T, Bonaldo MC, Goldenberg S. & de Souza W. (1989) A stereological study of the differentiation process in *Trypanosoma cruzi*. Parasitology Research, 75(7), 522–527. 2549536 10.1007/BF00931160

[R100] de Sousa MA (1999) Morphobiological characterization of *Trypanosoma cruzi* Chagas, 1909 and its distinction from other trypanosomes. Memorias do Instituto Oswaldo Cruz, 94(Suppl. 1), 205–210. 10677717 10.1590/s0074-02761999000700031

[R101] de Souza W. (1999) A short review on the morphology of *Trypanosoma cruzi*: from 1909 to 1999. Memorias do Instituto Oswaldo Cruz, 94(Suppl. 1), 17–36. 10677689 10.1590/s0074-02761999000700003

[R102] de Souza W, de Carvalho TU, Benchimol M. & Chiari E. (1978) *Trypanosoma cruzi*: ultrastructural, cytochemical and freeze-fracture studies of protein uptake. Experimental Parasitology, 45(1), 101–115. 352715 10.1016/0014-4894(78)90050-4

[R103] de Souza W, de Carvalho TM & Barrias ES (2010) Review on *Trypanosoma cruzi*: host cell interaction. International Journal of Cell Biology, 2010, 18. 10.1155/2010/295394 PMC2926652 20811486

[R104] de Souza W, Meza I, Martinez-Palomo A, Sabanero M, Souto-Padron T. & Meirelles MN (1983) *Trypanosoma cruzi*: distribution of fluorescently labeled tubulin and actin in epimastigotes. Journal of Parasitology, 69(1), 138–142. 6186802

[R105] de Souza W, Morgado-Diaz JA & Cunha-e-Silva NL (2008) Cell fractionation of parasitic protozoa. Methods in Molecular Biology, 425, 313–331. 10.1007/978-1-60327-210-0_25 18369906

[R106] de Souza W, Sant’Anna C. & Cunha-e-Silva NL (2009) Electron microscopy and cytochemistry analysis of the endocytic pathway of pathogenic protozoa. Progress in Histochemistry and Cytochemistry, 44(2), 67–124. 10.1016/j.proghi.2009.01.001 19410686

[R107] Steinert M. & Novikoff AB (1960) The existence of a cytostome and the occurrence of pinocytosis in the trypanosome, trypanosoma mega. The Journal of Biophysical and Biochemical Cytology, 8(2), 563–569. 10.1083/jcb.8.2.563 19866573 PMC2224929

[R108] Stevens JR (2008) Kinetoplastid phylogenetics, with special reference to the evolution of parasitic trypanosomes. Parasite, 15(3), 226–232. 10.1051/parasite/2008153226 18814685

[R109] Stevens JR (2014) Free-living bodonids and derived parasitic trypanosomatids: but what lies in between? Trends in Parasitology, 30(3), 113–114. 10.1016/j.pt.2014.01.002 24468209

[R110] Subramanya S. & Mensa-Wilmot K. (2010) Diacylglycerol-stimulated endocytosis of transferrin in trypanosomatids is dependent on tyrosine kinase activity. PLoS One, 5(1), e8538. 10.1371/journal.pone.0008538 PMC2796386 20049089

[R111] Theriot JA (2000) The polymerization motor. Traffic, 1(1), 19–28. 10.1034/j.1600-0854.2000.010104.x 11208055

[R112] Vatarunakamura C, Ueda-Nakamura T. & de Souza W. (2005) Visualization of the cytostome in *Trypanosoma cruzi* by high resolution field emission scanning electron microscopy using secondary and backscattered electron imaging. FEMS Microbiology Letters, 242(2), 227–230. 10.1016/j.femsle.2004.11.008 15621442

[R113] Vaughan S. & Gull K. (2015) Basal body structure and cell cycle-dependent biogenesis in *Trypanosoma brucei*. Cilia, 5, 5. 10.1186/s13630-016-0023-7 26862392 PMC4746817

[R114] Vickerman K. (1969) The fine structure of trypanosoma congolense in its bloodstream phase. The Journal of Protozoology, 16(1), 54–69. 10.1111/j.1550-7408.1969.tb02233.x 4896668

[R115] Vidal JC, Alcantara CL, de Souza W. & Cunha ESNL (2016) Loss of the cytostome-cytopharynx and endocytic ability are late events in *Trypanosoma cruzi* metacyclogenesis. Journal of Structural Biology, 196(3), 319–328. 10.1016/j.jsb.2016.07.018 27480509

[R116] Vizcaíno-Castillo A, Osorio-Méndez JF, Rubio-Ortiz M, Manning-Cela RG, Hernández R. & Cevallos AM (2019) *Trypanosoma cruzi* actins: expression analysis of actin 2. Biochemical and Biophysical Research Communications, 513(2), 347–353. 30961931 10.1016/j.bbrc.2019.04.007

[R117] Vizcaino-Castillo A, Osorio-Mendez JF, Ambrosio JR, Hernandez R. & Cevallos AM (2020) The complexity and diversity of the actin cytoskeleton of trypanosomatids. Molecular and Biochemical Parasitology, 237, 111278. 10.1016/j.molbiopara.2020.111278 32353561

[R118] Wallroth A. & Haucke V. (2018) Phosphoinositide conversion in endocytosis and the endolysosomal system. Journal of Biological Chemistry, 293(5), 1526–1535. 10.1074/jbc.R117.000629 29282290 PMC5798284

[R119] Wang W, Peng D, Baptista RP, Li Y, Kissinger JC & Tarleton RL (2021) Strain-specific genome evolution in *Trypanosoma cruzi*, the agent of Chagas disease. PLoS Path, 17(1), e1009254. 10.1371/journal.ppat.1009254 PMC7872254 33508020

[R120] Wootton EC, Zubkov MV, Jones DH, Jones RH, Martel CM, Thornton CA (2007) Biochemical prey recognition by planktonic protozoa. Environmental Microbiology, 9(1), 216–222. 10.1111/j.1462-2920.2006.01130.x 17227426

[R121] World Health Organization. (2015) Chagas disease in Latin America: an epidemiological update based on 2010 estimates. Weekly Epidemiological Record, 90(6), 33–43. 25671846

[R122] Yang ST, Kreutzberger AJB, Lee J, Kiessling V. & Tamm LK (2016) The role of cholesterol in membrane fusion. Chemistry and Physics of Lipids, 199, 136–143. 10.1016/j.chemphyslip.2016.05.003 27179407 PMC4972649

[R123] Zaman V, Howe J. & Ng M. (2000) Scanning electron microscopy of Chilomastix mesnili (Wenyon 1910) Alexieieff, 1912. Parasitology Research, 86(4), 327–329. 10.1007/s004360050051 10780743

[R124] Zapparoli D, Bertozzo TV, Alexandrino M, Sanches DF, Aires IN, Manzini S. (2022) Commercially acquired acai pulps contamination by *Trypanosoma cruzi*. International Journal of Food Microbiology, 363, 109508. 10.1016/j.ijfoodmicro.2021.109508 34971879

